# Supplementary material for: Mouse Spinal Cord Vascular Transcriptome Analysis Identifies CD9 and MYLIP as Injury-Induced Players
Source: Int J Mol Sci. 2023 Mar 29;24(7):6433. doi: 10.3390/ijms24076433 (PMC10094762; doi:10.3390/ijms24076433)
Supplement: Supplementary file 1 [file ijms-24-06433-s001.zip › ijms-2310385_Supplemental material.pdf]

Supplemental material

Table S1. RNA-seq sample details, including metrics considered in pre-processing.

Sample	Run	Days	Lesion	Condition	Decision	Library size
**M_3dpiSci_1_S_Run1_22**	1	3dpi	Sci	3dpi Sci	Kept	19.806.031
**M_3dpiSci2_S_Run1_4**	1	3dpi	Sci	3dpi Sci	Kept	13.671.058
**M_3dpiSci3_S_Run1_5**	1	3dpi	Sci	3dpi Sci	Kept	14.685.878
**M_3dpiSham1_S_Run1_1**	1	3dpi	Sham	3dpi Sham	Removed	493.364
**M_3dpiSham2_S_Run1_2**	1	3dpi	Sham	3dpi Sham	Removed	715.638
**M_3dpiSham3_S_Run1_3**	1	3dpi	Sham	3dpi Sham	Kept	13.698.148
**M_7dpiSci_1_S_Run1_9**	1	7dpi	Sci	7dpi Sci	Kept	11.585.719
**M_7dpiSci_3_S_Run1_8**	1	7dpi	Sci	7dpi Sci	Removed	3.564.291
**M_7dpiSham_1_S_Run1_6**	1	7dpi	Sham	7dpi Sham	Removed	50.047
**M_7dpiSham_2_S_Run1_7**	1	7dpi	Sham	7dpi Sham	Kept	15.958.959
**M_7dpiSham_3_S_Run1_10**	1	7dpi	Sham	7dpi Sham	Removed	2.025.102
**M_3dpiSham1_S_Run3_1**	3	3dpi	Sham	3dpi Sham	Kept	12.245.908
**M_7_DpiSci_1A_S_Run3_4**	3	7dpi	Sci	7dpi Sci	Kept	9.298.239
**M_7_DpiSham_1A_S_Run3_3**	3	7dpi	Sham	7dpi Sham	Kept	9.026.449
**M_3dpiSham2_S_Run4_2**	4	3dpi	Sham	3dpi Sham	Kept	9.537.205
**M_7_DpiSham_1A_S_Run4_7**	4	7dpi	Sham	7dpi Sham	Removed	1.564.290
**M_7dpiSham_1_S_Run4_3**	4	7dpi	Sham	7dpi Sham	Removed	710.086
**M_7dpiSham_1_S_Run5_1**	5	7dpi	Sham	7dpi Sham	Removed	371.243
**0dpisham1b_S_Run7_10**	7	0dpi	Sham	0dpi Sham	Kept	8.789.607
**0dpisham2b_S_Run7_11**	7	0dpi	Sham	0dpi Sham	Kept	7.804.849
**0dpisham3b_S_Run7_12**	7	0dpi	Sham	0dpi Sham	Removed *	8.351.209
**0dp1_sci1a_S_Run8_1**	8	0dpi	Sci	0dpi Sci	Kept	13.090.441
**0dp1_sci2_S_Run8_2**	8	0dpi	Sci	0dpi Sci	Kept	11.474.316
**0dp1_sci3a_S_Run8_3**	8	0dpi	Sci	0dpi Sci	Kept	13.340.809

* Sample with more than 7 million read counts but removed due a low number of profiled genes.

Table S2. qPCR Primers.

Name	Sequence
cd9_Fw	CGGTCAAAGGAGGTAGCAAGT
cd9_Rv	TGAGAGTCGAATCGGAGCCATA
mylip_Fw	CAGCTCCACTTTGAACAGCATC
mylip_Rv	CCACTCTATGCCGTAGTTCTCC
PPIA_Fw	TATCTGCACTGCCAAGACTGAGTG
PPIA_Rv	CTTCTTGCTGGTCTTGCCATTCC

Table S3. Western Blot Antibodies.

Antigen	Host	Dilution	Reference	Secondary	Dilution	Reference
**CD9**	Rabbit	1:1000	Abcam - ab92726	Goat anti-rabbit	1:3000	BioRad - 1706515
**MYLIP**	Rabbit	1:500	Invitrogen - PA5-106656	Goat anti-rabbit	1:3000	BioRad - 1706515
**GAPDH**	Mouse	1:1000	ThermoFisher Scientific - AM4300	Goat anti-mouse	1:4000	CAYMAN - 10004302

Table S4. Immunohistochemistry Antibodies e details.

Antigen	Host	Dilution	Reference	Retrieval	Blocking	Washing	Secondary	Dilution	Reference
**CD9**	Rabbit	1:100	Abcam - ab92726	Sodium Citrate Buffer, pH 6.0	1% BSA/0,3% Triton X-100 in PBS	0,1% Triton X-100 in PBS	Donkey anti-rabbit 488	1:400	Invitrogen - A21206
***αSMA**	Mouse	1:100	Sigma - C6198				NA - Cy3 conjugated antibody		
**CD31**	Goat	1:100	RD Systems - AF3628				Donkey anti-goat 647	1:500	Invitrogen - A21447
**MYLIP**	Rabbit	1:100	Invitrogen - PA5-106656	NA	2% BSA/0,2% Triton X-100 in PBS	0,2% Triton X-100 in PBS	Donkey anti-rabbit 488	1:500	Invitrogen - A21206
**CD13**	Rat	1:100	BioRad - MCA2183T				Donkey anti-rat 647	1:500	712-605-153
**CD31**	Goat	1:100	RD Systems - AF3628				Donkey anti-goat 568	1:500	Invitrogen - A11057

* CD13 was used as a gold standard immunostaining to detect pericytes. CD13 however does not work with the antigen retrieval protocol necessary to detect CD9. Given this, we used a compatible antibody for pericytes detection (αSMA).
